# Impact of caregiver’s eHealth literacy, financial well-being, and mental health on quality of life of pediatric patients with osteogenesis imperfecta

**DOI:** 10.1186/s12955-023-02148-4

**Published:** 2023-07-07

**Authors:** Richard Huan Xu, Liling Zhu, Rongjia Sun, Sainan Zou, Dong Dong

**Affiliations:** 1 Department of Rehabilitation Sciences, The Hong Kong Polytechnic University, Hong Kong SAR, China; 2 JC School of Public Health and Primary Care, The Chinese University of Hong Kong, Hong Kong SAR, China; 3 The Illness Challenge Foundation, Beijing, China; 4 Department of Intensive Care Unit, The Sith Affiliated Hospital of Sun Yat-Sen University, Guangzhou, China

**Keywords:** Health-related quality of life, Osteogenesis imperfecta, Caregiver, eHealth literacy, Financial well-being, Mental health

## Abstract

**Objective:**

This study assesses the association between health-related quality of life (HRQoL) for pediatric patients with osteogenesis imperfecta (OI) and their caregivers’ eHealth literacy (eHL), financial well-being, and mental health along with the impact of eHealth literacy on the financial well-being and mental health of OI caregivers.

**Methods:**

Participants were recruited from a member pool of two OI patient organizations in China. Information about patients’ HRQoL and their caregivers’ eHL, financial well-being, and mental health was collected. Structure equation modeling (SEM) was used to estimate the relationship between the measures. The robust weighted least square mean and variance adjusted estimator was used. Three criteria, the comparative fit index, the Tucker-Lewis index, and the root mean square error of approximation, were used to evaluate the goodness-of-fit of the model.

**Results:**

A total of 166 caregivers completed the questionnaires. Around 28.3% indicated that pediatric OI patients experienced problems related to mobility, and 25.3% reported difficulty doing usual activities. Around 52.4% of caregivers reported that their care receivers have some emotional problems while 8.4% reported that their care receivers have “a lot of” emotional problems. ‘Some problems’ on all dimensions on EQ-5D-Y was the most frequently reported health state (13.9%), and around 10.0% have no problems on all dimensions on EQ-5D-Y. Caregivers tended to show a significantly high eHL, financial well-being, and mental health when their care receivers reported no problems with usual activities and emotions. The SEM demonstrated a significant and positive relationship between eHL, financial well-being, and mental health.

**Conclusion:**

OI caregivers with high eHL reported satisfactory financial well-being and mental health; their care receivers rarely reported living with poor HRQoL. Providing multicomponent and easy-to-learn training to improve caregivers’ eHL should be highly encouraged.

## Introduction

Osteogenesis imperfecta (OI) represents a group of rare genetic disorders that mainly affect the bones. It is characterized by increased bone fragility and reduced bone mass, which increase one’s risk of a fracture [1]. Globally, OI is estimated to affect approximately 1 in 10,000–20,000 people [2]. Clinical evidence demonstrates that due to recurrent fractures and bone deformity, pediatric OI patients, starting from early stages of their life, typically require periodic medical exams, orthopedic surgery, drug therapy, and physiotherapy that result in an impaired health-related quality of life (HRQoL) and lead to multiple challenges for the caregivers, particularly related to their health, emotions, and finances.

There is a lack of official registry data on OI in China. According to a report by ‘*Chinadolls*’, one of the largest OI patient associations in China, there were an estimated 100,000 OI patients nationwide in 2013. Currently, most studies on OI in China focus on patients' clinical characteristics and genotype–phenotype correlations, evidence about their HRQoL is limited. For example, a study conducted in Northern China indicated that HRQoL was significantly impaired in adult OI patients, and patients with more severe OI had poorer HRQoL outcomes [3]. Another study targeted both adult and child OI patients, confirming that from the patients' own perspective, subjective QoL is the most important matter in their lives [4]. However, there is, currently, no research assessing the associations between pediatric OI patients' HRQoL and their caregivers' health and socioeconomic status, which has been confirmed as an important predictor of health outcomes in children [5].

Although health and social care support are available for patients with rare diseases (e.g., OI), in most industrial countries, the support systems are often identified as inadequate [6]. Caring for pediatric OI patients is a life-long and complex process. The changing dynamics and routines as well as extra family duties are likely to result in conflict between caregivers and may lead to diminished mental health [7]. Qualitative evidence shows that parental caregivers of pediatric OI patients tend to suffer from increased stress and poorer QoL [7, 8]. Moreover, research also indicates that pediatric OI patients living with additional chronic disease can trigger high stress levels in caregivers [9]. Although these findings offer meaningful information about the burden of care for OI patients, there is a paucity of quantitative evidence about the relationship between caregivers’ mental health and the care receivers’ HRQoL in a large sample of the OI population.

The impact of financial burdens on poor physical and mental health for OI families are discussed in several qualitative studies [7, 10–12]. However, unlike financial burden, which is related to the cost of medical care, financial well-being is a concept that reflects the perception of being able to sustain current and anticipated desired living standards and financial freedom [13]. While this concept has been widely studied in business, it has attracted less attention in health research. Given that financial concerns are a major source of distress and financial help is a main area of need for the parents of pediatric patients with a rare disease [14], financial well-being can be a useful measure to help policymakers assess not only the effectiveness of an intervention but to plan future support strategies. However, models of patient financial well-being cannot be applied across different diseases without considering their specific medical conditions and socioeconomic characteristics. Considering that pediatric OI patients usually need life-long treatment, there is a high risk of financial unsustainability for their caregivers, and the feeling of having a secure financial future is essential for them to make choices and enjoy life [15]. Although the literature has already established the detrimental relationship between poor financial conditions and patients’ health [16], evidence about financial well-being and its impacts to OI caregivers is lacking.

Although information is always scarce in the field of rare diseases [17], the Internet has increasingly become a valuable source for individuals with rare disorders, including OI, to manage their health in recent years. Castro et al. found that some OI caregivers tend to use Internet-based technologies to broaden their access to information to relieve feelings of distress and the experience of social isolation [18]. However, other research has indicated that most OI caregivers have experienced difficulties obtaining information regarding treatment, financial assistance, and social support [12]. It is challenging for them to decipher what is true and accurate and what is not in the infinite amount of online information. OI caregivers’ eHealth literacy (eHL), that is the ability to appraise health information from electronic sources and apply the knowledge to addressing health problems, is unknown. Research on the use of web-based information for caregivers of pediatric patients with rare diseases is an emerging field; and several studies have demonstrated that patients with rare diseases show insufficient eHL to appraise the quality of web-based information [19–21]. However, evidence about the relationships between pediatric OI patients’ HRQoL and their caregivers’ eHL, financial well-being, and mental health as well as the impact of caregiver’s eHL on mental and financial well-being is absent. Given the Internet is used substantially in health information-seeking, eHL is vital for caregivers to have the reliable resources to optimize their caregiving and improve finanical capability and well-being. Therefore, this study aims to preliminarily assess two hypotheses. First, the association between OI patients’ low HRQoL and, separately, their caregivers’ low eHL, financial well-being, and mental health. Second, the positive relationship between high eHL, financial well-being, and mental health in OI caregivers.

## Methods

### Survey design and participants

A web-based and cross-sectional survey was conducted to collect data between May and December 2021. Participants were recruited from the member pool of two OI patient organizations in China. Patients who met these two criteria were selected: 1) aged between 8 and 18 years and 2) have a family history of OI or clinical presentation (with at least one symptom or sign of OI). Primary caregivers, defined as someone who has faced the duty of taking care of pediatric OI patients, of eligible patients (8–17 years) were invited to complete a questionnaire about the patients’ HRQoL, their eHL, financial well-being, and mental health. Those who could not read or understand Chinese were excluded from the survey.

The research team collaborated with the managers of the patient organizations by sending the survey invitation and related information to all members via their internal social network. The eligibility of participants was examined based on the information provided to the patient organizations. A total of 175 primary caregivers of pediatric OI patients contacted the research team. After examination, all pediatric OI patients were eligible to participate in the survey and their caregivers were requested to join an online survey group. The participants were informed about the guidelines for completing the online questionnaire. Information about their demographics, socioeconomic status, and other characteristics of interest was collected. In total, 166 caregivers completed the questionnaire (response rate = 94.9%). The study proposal and informed consent were approved by the Survey and Behavioral Research Ethics Committee of the Chinese University of Hong Kong (Ref ID: SBRE-19–793). Written informed consent was obtained from all participants.

### Measures

#### Background information

Caregiver’s background information about their relationship to the patient, educational level, age, family registry, employment, marital status, annual family income, and health status was collected.

#### HRQoL

The proxy version of EuroQol five-dimension measure youth (EQ-5D-Y) questionnaire was used to measure OI patients’ HRQoL. The EQ-5D-Y has five dimensions (mobility, looking after myself, doing usual activities, having pain or discomfort, and feeling worried, sad, or unhappy). Each dimension has three levels: no problems, some problems, and a lot of problems [22]. The best health state is “11,111,” which means no problem on all five dimensions, whereas the worst health state is “33,333,” which means a lot of problems on all five dimensions.

#### eHealth literacy

The eHealth literacy scale (eHEALS) was used to measure people’s ability to search, analyze, and use web-based information to manage their health [23]. The eHEALS is comprised of eight items, and the sum score ranges between 0 and 40; the higher the score the better the eHL.

#### Financial well-being

InCharge Financial Distress/Financial Well-Being Scale (IFDFW) was used to assess financial well-being in this study [24]. It aims to measure the latent construct representing responses to one’s financial state on a continuum ranging from the lowest to highest level of financial wellbeing. Respondents are asked to rate their financial status on a scale ranging between 0 and 10, where a high score indicates a low financial distress.

#### Mental Health

Mental health was measured by the Short Warwick-Edinburgh Mental Well-being Scale (SWEMWS), which aims to monitor mental health and well-being; and evaluate the effectiveness of projects and policies to improve it [25]. It includes seven statements with a five-response category. A higher score indicates better mental health status.

### Statistical analysis

Descriptive analysis was used to describe the caregivers’ background characteristics. Continuous and categorical variables are presented using a mean (standard deviation) and number (percentage), respectively. Analysis of variance was used to assess whether the eHEALS, IFDFW, and SWEMWS discriminate the different risk groups.

Structural equation modeling (SEM) was used to analyze the hypothesized relationship between financial well-being, mental health, and eHL, and to test the model fit. The robust weighted least square mean and variance adjusted estimator, which assumes non-normally distributed variables and provides the best option for modeling categorical or ordered data, was used. Three criteria were used to evaluate the goodness-of-fit of the model. They include 1) the comparative fit index (CFI) where values above 0.9 show a good model fit; 2) the Tucker-Lewis index (TLI) where values above 0.9 indicate a good model fit; and (3) the root mean square error of approximation (RMSEA) where values less than 0.08 are equal to a “close fit” [26]. R software was used to perform all data analysis. Statistical significance is based on a *p*-value ≤ 0.05.

## Results

### Caregivers’ characteristics

Table 1 presents the background characteristics of the participants. Approximately 97% are parental caregivers, 58.4% are female, and more than 60.0% have completed secondary education or higher. The mean age of caregivers is 39.6 years and around 63.6% indicated that their family annual income could not cover daily expenses.

**Table 1:** Participants’ background characteristics

	**n**	**%**
Relationship with patient		
Parents	160	97
Grand parents	6	3
Gender		
Male	69	41.6
Female	97	58.4
Educational level		
Primary or below	49	29.5
Secondary	98	59.1
Tertiary or above	19	11.4
Family registry		
Urban	47	28.3
Rural	119	71.7
Employment		
Active	89	53.6
Inactive	77	46.4
Marital status		
Married	150	90.4
Single	3	1.8
Divorce/widow(er)	13	7.8
Perceived family income		
Cannot meet daily cost	105	63.6
Can meet daily cost	58	35.2
Surplus after meeting daily cost	2	1.2
Chronic condition		
Yes	68	41
No	98	59
Age [mean (standard deviation), range]	39.6 (7.2)	23 ~ 74

### HRQoL for OI patients

**Fig. 1:**
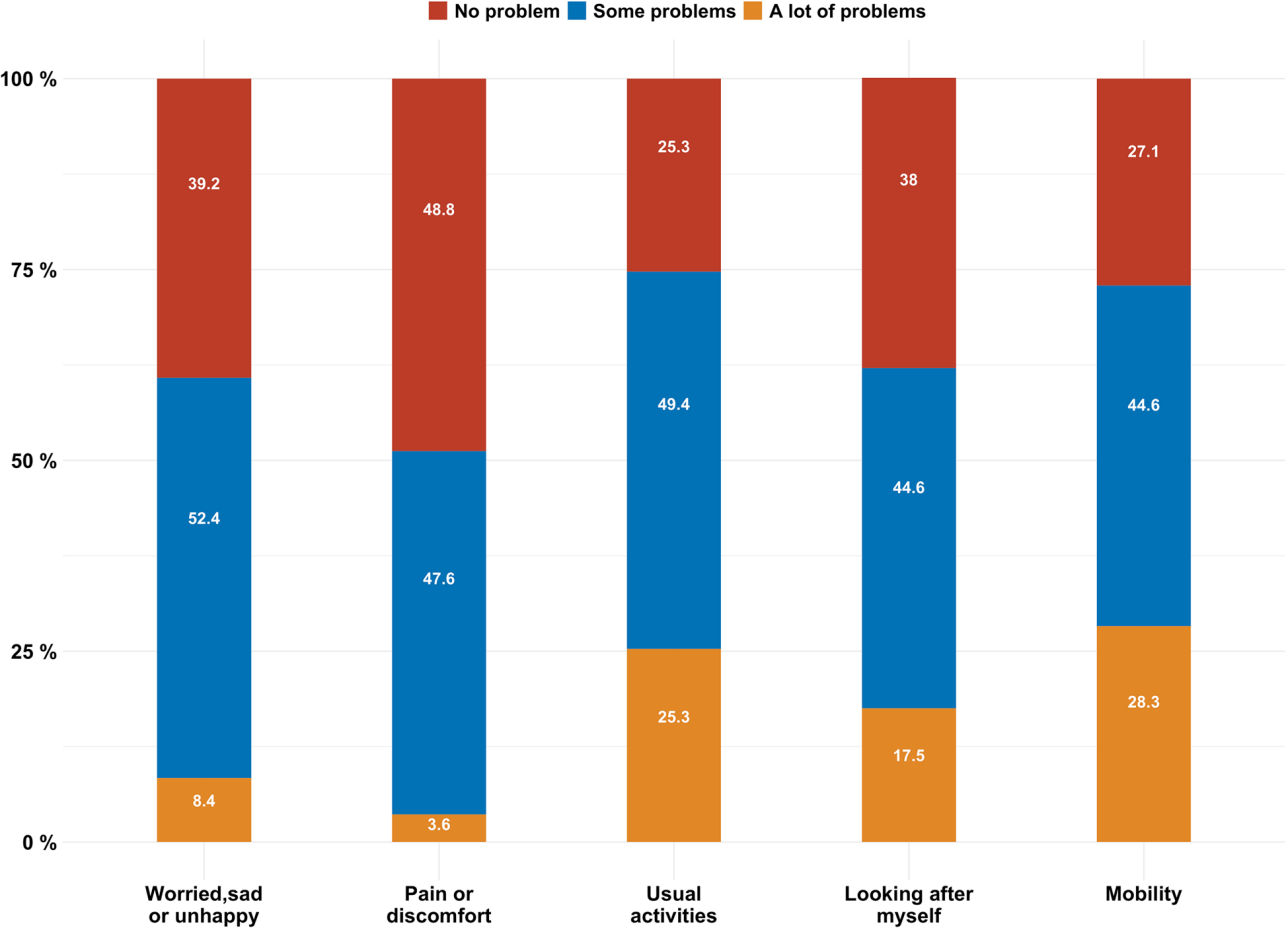
Response on EQ-5D-Y dimensions

Patient’s HRQoL is described by the EQ-5D-Y descriptive system. Around 28.3% and 25.3% of caregivers indicated their children have “a lot of” problems regarding mobility and doing usual activities, respectively. Around 52.4% of caregivers reported that their care receivers have some emotional problems while 8.4% reported that their care receivers have “a lot of” emotional problems. Approximately half of caregivers indicated that their care receivers have no problems on pan/discomfort Fig. 1.

**Fig. 2:**
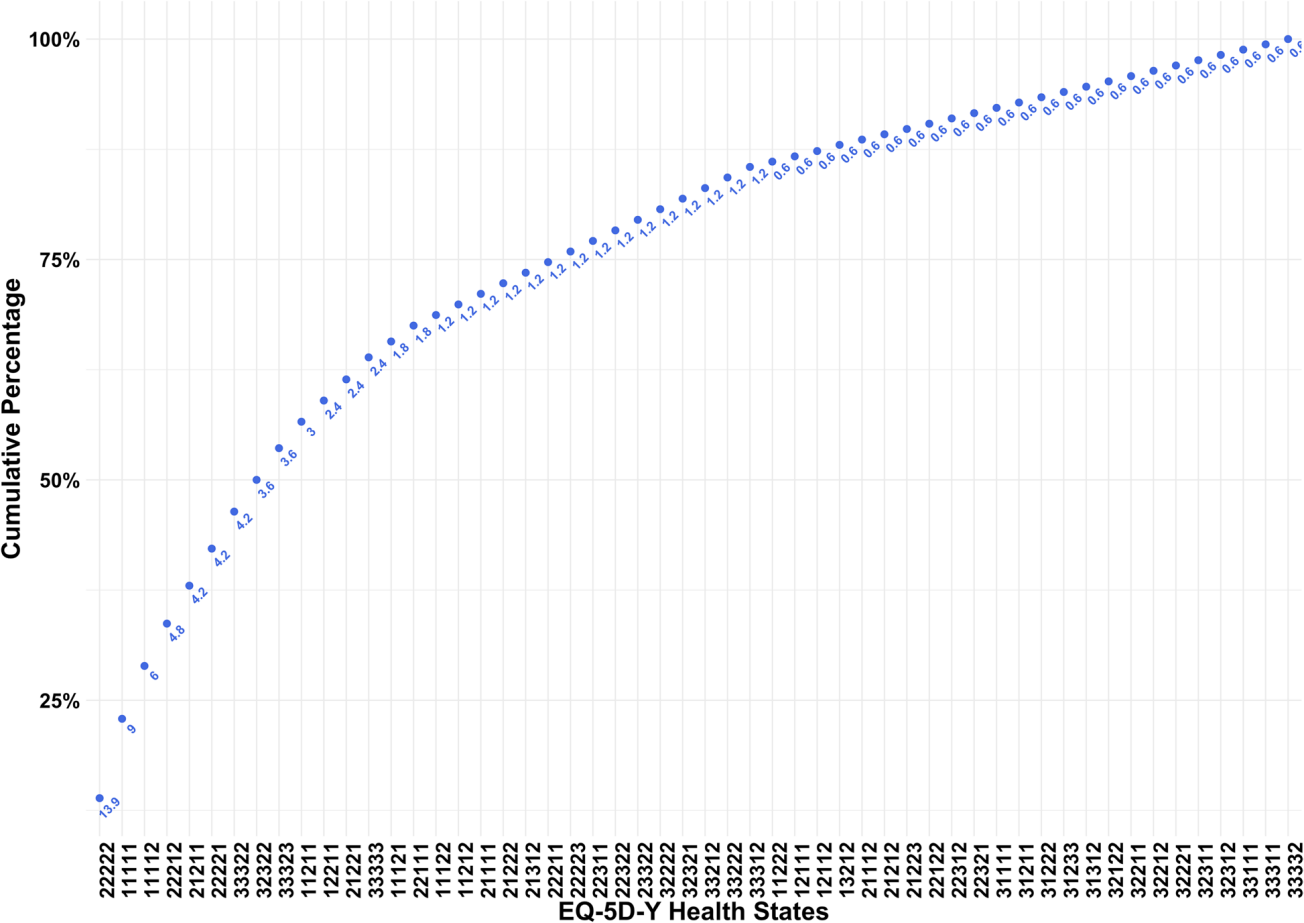
Cumulative percentage of EQ-5D-Y health states

Figure 2 presents that health state “22,222” (some problems on all five EQ-5D-Y dimensions) is the most frequently reported health state (13.9%) for the respondents. Most pediatric patients reported experiencing problems (level 2 or 3 on that dimension) with performing their usual activities. Among the top ten health states described by the EQ-5D-Y, five of them showed that patients experienced some problems in performing their usual activities (e.g., “22,222” and “22,212”). Three of them showed that patients experienced many problems in performing their usual activities (e.g., “33,322” and “32,322”). Approximately 9% of the caregivers indicated their care receivers have no problems on all dimensions on EQ-5D-Y (the second health state – “11,111”). In addition, health state “33,333” (a lot of problems on mobility, looking after myself, doing usual activities, having pain or discomfort, and feeling worried, sad, or unhappy) is the worst health state reported by the caregivers and around 2.4% of patients are experiencing with it. The health state "33,332" (a lot of problems with mobility, looking after oneself, doing usual activities, experiencing pain or discomfort, and some problems with feeling worried, sad, or unhappy) was reported by the fewest respondents, accounting for approximately 0.5%.

### Patients’ HRQoL and caregivers’ eHL, financial well-being, and mental health

Caregivers show a significantly high eHL and financial well-being, and mental health when their care receivers reported no problems on usual activities and emotions. Caregivers indicated a high financial well-being when care receivers reported no problems “looking after self” and “having pain/discomfort.” Caregivers’ high eHL and financial well-being is significantly associated with patients with a low frequency of bone fractures and fewer complications. Additionally, caregivers who reported that their family income could meet their daily expenses had significantly higher eHL, financial well-being, and mental health compared to those who reported that their family income could not meet their daily expenses (Table 2).

**Table 2:** Relationship between eHealth literacy, financial well-being, and mental health and HRQoL

	**n**	**eHealth literacy**		**Financial well-being**		**Mental health**	
		Mean(sd)	*p*-value	Mean(sd)	*p*-value	Mean(sd)	*p*-value
**EQ-5D-Y dimensions**							
Mobility							
*No problem*	45	29.6(5)	0.11	64.8(13.9)	0.11	21.9(4.2)	0.19
*Some problems*	121	28.1(5.3)		61(13.9)		20.9(4.3)	
Looking after self							
*No problem*	63	29.5(4.7)	0.07	65.9(13.6)	0.03	21.7(3.9)	0.21
*Some problems*	103	28(5.5)		60.3(13.9)		20.9(4.5)	
Doing usual activity							
*No problem*	42	30(4.8)	0.04	65.4(12.9)	0.008	22.5(4.2)	0.02
*Some problems*	124	28.1(5.3)		58.9(15.7)		20.7(4.2)	
Having pain/discomfort							
*No pain/discomfort*	81	29.4	0.06	67(11)	0.002	21.6(3.9)	0.21
*Some pain/discomfort*	85	27.9		60.3(15.8)		20.8(4.6)	
Feeling worried, sad, or unhappy							
*No*	65	29.6(4.9)	0.04	67.1(11)	< 0.001	22.4(4.7)	0.004
*Some worry, sad or unhappy*	101	28(5.4)		58.5(16.3)		20.4(3.8)	
**Frequency of bone fracture per year**							
≤ 30 times	70	30(5.6)	0.004	65.5(12.1)	0.03	21.7(4.4)	0.18
≥ 31 times	94	27.6(4.8)		61(15.8)		20.8(4.2)	
**Number of complications**							
≤ 1	69	29.9(4.5)	0.007	69.6	0.04	21.6	0.21
2–3	76	28(5.8)		64.5		21.2	
≥ 4	20	26.2(4.7)		61.2		19.7	
**Perceived family income**							
Cannot meet daily cost	105	27.7(5.4)	0.003	52.8(13.5)	< 0.001	20.2(3.8)	< 0.001
Can meet daily cost	60	30.2(4.6)		70.1(9.7)		22.8(4.6)	

**Fig. 3:**
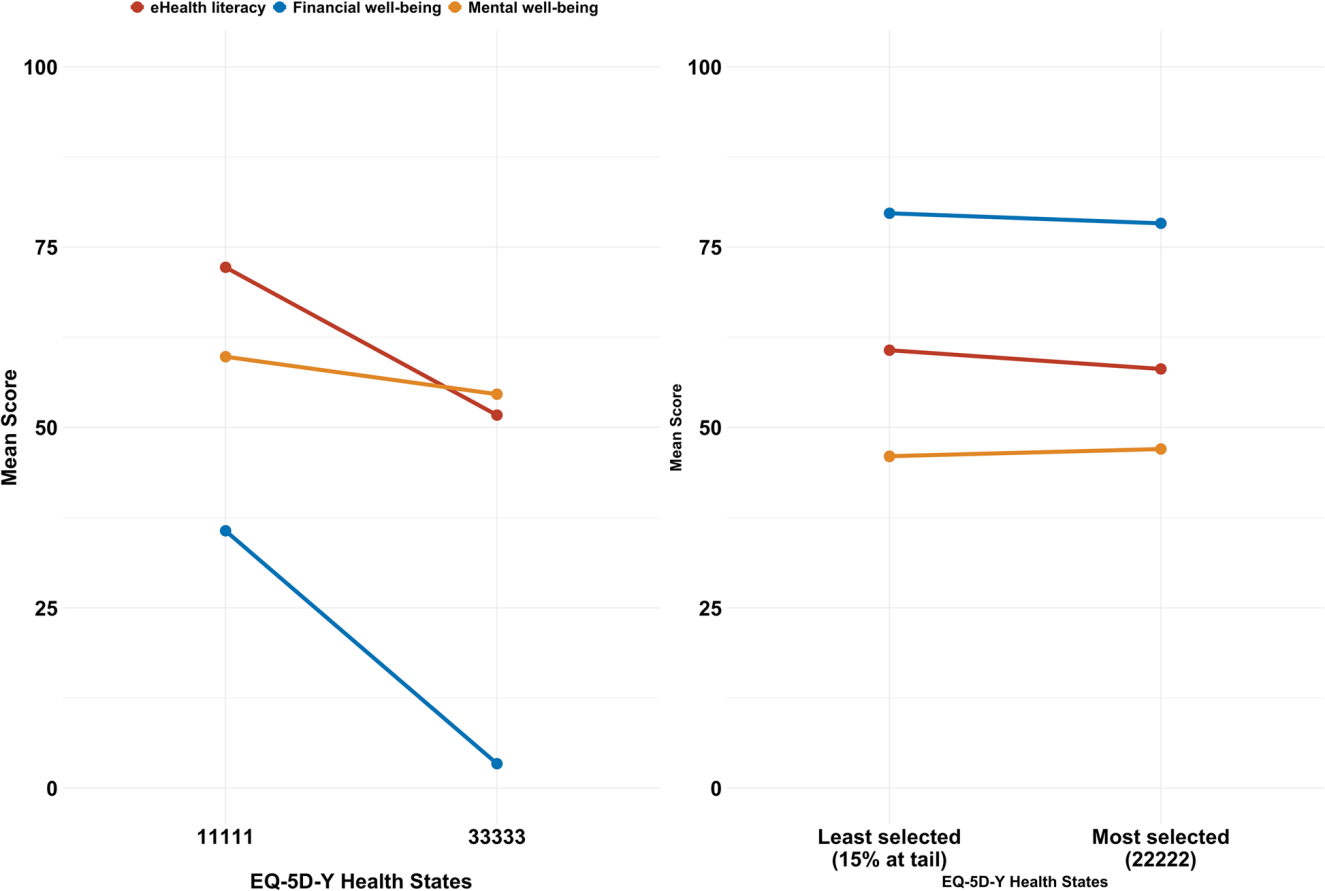
Mean score of three measures stratified by health states

Figure 3 shows that caregivers reported a higher eHL, financial well-being, and mental health when a patient is in the best health state (“11,111”, no problems on all dimensions of the EQ-5D-Y), compared to those with the worst health state (“33,333”, a lot of problems on all dimensions of the EQ-5D-Y). However, the difference in the financial well-being and mental health between the most selected (“22,222”, some problems on all five dimensions of the EQ-5D-Y) and least 15% selected EQ-5D-Y health states is negligible and statistically insignificant. Caregivers of patients with the most selected health state reported a lower eHL than what patients with the least 15% selected health state reported.

### Correlations between eHL, financial well-being, and mental health

Table 3 demonstrates that all measures show a high reliability in our sample, and the associations are positive and statistically significant. After normalization, caregivers reported a lower financial well-being (Mean_IFDFW_ = 24.2) than mental health (Mean_SWEMWS_ = 48.5) and eHL (Mean_eHEALS_ = 62).

**Table 3:** Reliability, descriptive statistics, and correlations of measures

	α	Normalized mean (0 ~ 100)	Median	SD	Range	Correlations		
						1	2	3
1. eHEALS	0.95	62	63.3	17.5	0 ~ 100	-	-	-
2. IFDFW	0.91	24.2	20.1	21	0 ~ 100	0.29^***^	-	-
3. SWEMWS	0.84	48.5	44.4	16.2	0 ~ 100	0.31^***^	0.46^***^	-

^***^ *p* < 0.001

The standardized path coefficients and the model fit for the SEM are demonstrated in Table 4. The SEM model fits the data well with CFI = 0.937, TLI = 0.929, and RMSEA = 0.066. Factor loadings range between 0.465 and 0.925. The standardized solution for the paths of the SEM shows a significant and direct effect on mental health. The higher the financial well-being, the better the mental health (*β* = 0.406, *p* < 0.001). Mental health is positively correlated with eHL (*β* = 0.241, *p* = 0.002), indicating that the higher the eHL, the better the mental health of caregivers. A high eHL can lead to a significantly satisfactory financial well-being (*β* = 0.288, *p* < 0.001).

**Table 4:** Factor loadings, mean, and path coefficients of the SEM

Scale	Item	Mean (SD)	Standardized Loading	*p*-value
**Factor loadings**				
Mental health	M1	3.68(0.71)	0.624	< 0.001
Mental health	M2	3.57(0.74)	0.681	< 0.001
Mental health	M3	3.66(0.78)	0.538	< 0.001
Mental health	M4	3.64(0.77)	0.644	< 0.001
Mental health	M5	3.6(0.79)	0.689	< 0.001
Mental health	M6	3.54(0.79)	0.722	< 0.001
Mental health	M7	3.48(0.78)	0.817	< 0.001
eHealth literacy	E1	3.43(0.77)	0.669	< 0.001
eHealth literacy	E2	3(0.95)	0.855	< 0.001
eHealth literacy	E3	3.28(0.89)	0.87	< 0.001
eHealth literacy	E4	2.25(0.93)	0.85	< 0.001
eHealth literacy	E5	3.07(0.73)	0.872	< 0.001
eHealth literacy	E6	3.16(0.8)	0.834	< 0.001
eHealth literacy	E7	3.18(0.85)	0.876	< 0.001
eHealth literacy	E8	3.18(0.85)	0.844	< 0.001
Financial well-being	F1	3.14(2.21)	0.765	< 0.001
Financial well-being	F2	3.42(2.41)	0.764	< 0.001
Financial well-being	F3	2.73(1.74)	0.843	< 0.001
Financial well-being	F4	2.71(1.94)	0.865	< 0.001
Financial well-being	F5	3.06(2.24)	0.692	< 0.001
Financial well-being	F6	3.27(2.79)	0.465	< 0.001
Financial well-being	F7	2.87(2.34)	0.759	< 0.001
Financial well-being	F8	3.04(2.06)	0.925	< 0.001
**Path**			*β*	*p*-value
Financial well-being → Mental health			0.406	< 0.001
eHealth literacy → Mental health			0.241	0.002
eHealth literacy → Financial well-being			0.288	< 0.001
**Model performance**	value			
RMSEA	0.066			
CFI	0.937			
TLI	0.929			

## Discussion

This study confirms that there are statistically significant associations between pediatric OI patients’ increased HRQoL and their caregivers’ high eHL, financial well-being, and mental health, but the correlation between HRQoL and financial well-being is stronger than the eHL and mental health. Additionally, high eHL is associated with satisfactory financial well-being, and good mental health in OI caregivers. Although caring for OI patients can generate emotional distress and extended care can be a risk factor for financial burden, our findings show that if caregivers are equipped with a high ability to seek and use web-based health-related information, the negative impact of mental distress and financial hardship might be mitigated. Furthermore, the majority of caregivers indicate that OI patients encounter either some or more problems doing their usual activities and thus their inability to perform usual activities might be an important source of OI caregivers’ emotional distress and financial hardship, which may need further exploration.

Despite its importance, eHL, measured using a validated scale, has not been examined in OI caregivers and has rarely been reported in pediatric patients [20]. In this study, we found that OI patients indicate no problem doing usual activities, few mental health problems, infrequent bone fractures, and few complications when their caregivers have a high eHL. However, the level of eHL (unnormalized mean = 28.6) for OI caregivers in this study is relatively lower than what is reported by the general Chinese population [23]. Previous studies have reported difficulties finding reliable web-based information. For example, Kasparian et al. found that over half of the parents of children with coronary heart disease reported difficulties accessing and using eHealth resources [20]. Another German study demonstrated that less than 10% of the patients with rheumatology stated that they knew some useful websites/mobile rheumatology apps, and reported a lower eHEALS mean (26.3) compared to the mean in the present study [27]. However, there is a paucity of empirical evidence about the relationship between patients’ HRQoL and caregivers’ eHL. Although caregivers with high eHL are more likely to find high-quality, evidence-based, and rigorously evaluated resources via the Internet to improve their care receiver’s HRQoL than those with low eHL, most research focuses on studying elderly people with dementia [28–30]. Our study provides quantifiable information that improving the caregivers’ eHL can be beneficial for enhanced HRQoL of OI patients. However, a relatively low eHL highlights the important need for structured eHL guidance and patient-adapted information for the caregivers of patients with rare diseases to improve their ability to use web-based resources.

OI patients’ HRQoL show a strong correlation with their caregivers’ financial well-being. This is not reported by previous research as most either assess the association between patients’ HRQoL and the family’s financial crisis [31–33] or between the caregivers’ financial burden and their own quality of life [34, 35]. In this study, regarding the financial well-being, caregivers are most worried about their inability to meet normal monthly living expenses (F4 in Table 4). This worry might not be only related to their insufficient actual income, but also to their money management skills, confidence in their ability to deal with financial obligations, and anticipation of their children’s medical condition [36]. OI is a rare disorder that can be either life-threatening or chronically debilitating, which means caregivers may face either immediate financial crisis or long-term financial hardship. Research on rare diseases exhibits that, except for providing direct or one-off financial support [37], our health and social systems must be resilient, adapt effectively to patients’ changing conditions, help with the significant challenges using limited resources, and reduce the negative impact of uncertainties because of the loss of purpose and hope.

Taking care of a child with rare diseases is mentally challenging. For example, Da Paz and Wallander showed that parenting a child with autism spectrum disorder is associated with enhanced distress and mental health problems [38]. Wu et al. found that family caregivers experience substantial stress and an overwhelming burden when caring for patients with epidermolysis bullosa [39]. Another Dutch study demonstrated that low mental health is reported by mothers of patients with spinal muscular atrophy, and leads to their restricted participation in social/leisure activities [40]. In this study, OI caregivers’ mental health was significantly affected by their children’s mental (worry) and physical HRQoL (doing usual activities). Given that a significant contribution of high eHL to better mental health was observed in this study, education, and training for improving the ability to seek and use reliable web-based information regarding peer and professional psychological support in rare disease caregivers should be highly encouraged.

We found that most pediatric OI patients face problems doing their usual activities, including work, study, homework, and leisure activities. This is consistent with previous findings. For instance, Song et al. in their study reported that children with OI scored lower on the domain of school functioning of PedsQL than the other domains [3]. Tsimicalis et al. demonstrated that fear of fractures can influence the choice of employment and leisure activities in OI patients [41]. Another UK study reported that over half of OI adults reported problems with the ability to carry out usual activities, using the EQ-5D adult version [42]. A direct outcome of OI children’s inability to carry out their usual activities for their caregivers is that they have less time to participate in social or leisure activities, which is also true for caregivers of other childhood onset rare neurodevelopmental illnesses [43]. This leads to social isolation to some extent, and may contribute to being more anxious and depressed. The Internet might be a useful way to strengthen and maintain social ties for caregivers, but only when they are equipped with adequate eHL.

## Limitations

This study has several limitations that must be addressed. First, the study sample was recruited from a volunteer pool where participants might be in better health compared to non- volunteers; thus, the sample may have selection bias. Second, all data were collected via a web-based survey; participants who were not familiar with such surveying methods might have provided inappropriate answers, which may have led to information bias. Third, given that all data were self-reported by OI caregivers, no clinical information, for example, OI types, were collected, which may affect the generalizability of our findings.

## Conclusion

This study demonstrates a statistically significant relationship between pediatric OI patients’ enhanced HRQoL and their caregivers’ high eHealth literacy, financial well-being, and mental health. Caregivers who show a high ability to navigate web-based information to solve health-related problems tend to report a high financial well-being and mental health. Our findings fill the research gap that no study investigating the role of eHL in caregivers of pediatric rare disease patients has done thus far, by quantifying the importance of eHL in improving their well-being. Additionally, caregivers show an inadequate eHL and one that is lower than the Chinese general population. Hence, education and training for caregivers and optimizing Internet use in caring for pediatric OI patients should be encouraged. Given that OI caregivers reported extremely high financial stress, providing multicomponent and easy-to-use Internet-based financial support should be considered.

## Data Availability

The datasets generated during and/or analyzed during the current study are available from the corresponding author on reasonable request.
